# The prevalence and prognostic value of systemic inflammation in good performance status patients with advanced, inoperable non‐small cell lung cancer receiving palliative radiotherapy: Comparison of composite ratios and cumulative scores

**DOI:** 10.1002/cam4.70139

**Published:** 2024-08-20

**Authors:** Josh McGovern, Fraser O'Rourke, Sarah Will, Hanh Thi Ngoc Nguyen, Elise Cranfield, Charlotte Maseland, Nicholas MacLeod, John D. Maclay, Barry J. Laird, Ross D. Dolan, Donald C. McMillan

**Affiliations:** ^1^ Academic Unit of Surgery University of Glasgow Glasgow UK; ^2^ Department of Oncology Beatson West of Scotland Cancer Centre Glasgow UK; ^3^ Department of Respiratory Medicine Glasgow Royal Infirmary Glasgow UK; ^4^ Institute of Genetics and Molecular Medicine University of Edinburgh Edinburgh UK

**Keywords:** cancer, inflammation, NSCLC, survival

## Abstract

**Introduction:**

The present study sought to examine the relationships between systemic inflammatory composite ratios/cumulative scores, magnitude of systemic inflammatory response (SIR) and survival in good performance status patients (ECOG‐PS 0/1) with advanced NSCLC receiving palliative radiotherapy.

**Methods:**

Systemic inflammatory composite ratios/cumulative scores included the neutrophil‐lymphocyte ratio (NLR), platelet‐lymphocyte ratio (PLR), lymphocyte‐monocyte ratio (LMR), C‐reactive protein, (CRP)‐albumin ratio (CAR), neutrophil‐ lymphocyte score (NLS), platelet‐lymphocyte score (PLS), lymphocyte‐monocyte score (LMS), neutrophil‐platelet score (NPS), modified Glasgow prognostic score (mGPS). The magnitude of SIR was determined by serum CRP concentration, with a median CRP concentration of >10 m mg/L considered to be systemically inflamed. Relationships between systemic inflammatory composite ratios/ cumulative scores and clinicopathological characteristics were examined using chi‐square analysis. Relationships between overall survival (OS) and systemic inflammatory composite ratios/ cumulative scores were examined using cox regression analysis.

**Results:**

479 patients were included. 48% (*n* = 231) of patients were male and 70% (*n* = 338) were ≥65 years of age. 29% (*n* = 140) patients were ECOG‐PS 0 and 71% (*n* = 339) were ECOG‐PS 1. 98% (*n* = 469) of patients died during follow‐up. The median survival was 5 months (2–11). A similar prevalence of systemic inflammation was noted across the various ratios/scores (NLR >3 68%; LMR <2.4 65%; PLR >150 70%; CAR >0.20 83%; NLS ≥1 66%; LMS ≥1 71%; NPS≥1 50%; PLS≥1 60% and mGPS≥1 75%). Despite not considered to be systemically inflamed, an NLR <3, LMR ≥2.4, PLR ≤150, NLS 0, LMS 0, NPS 0 and PLS 0 all had a median CRP concentration of >10 mg/L. When adjusted for ECOG‐PS, CAR>0.40 (*p* < 0.001) and mGPS 2 (*p* < 0.05) remained significantly associated with OS.

**Conclusion:**

Liver‐based measures of systemic inflammation (CAR and mGPS) appear more reliable for the quantification of the magnitude of SIR and have prognostic value in patients with advanced NSCLC.

## 1 INTRODUCTION

Non‐small cell lung cancer (NSCLC) is responsible for approximately 75% of lung cancer cases.^1^ Contemporary studies suggest that most patients with NSCLC are diagnosed with locally advanced or metastatic disease.^2^ As such, survival outcomes for NSCLC remain poor in comparison to other cancer subtypes.^1^ While treatment options for advanced, inoperable NSCLC were historically limited to palliative chemotherapy and/or radiotherapy,^3^, ^4^ novel target therapies and immunotherapy may have the potential to improve survival outcomes.^5^, ^6^ Therefore, the identification of prognostic factors that may predict likely outcome in patients with advanced NSCLC remains of interest.^7^

The Eastern Cooperative Oncology Group (ECOG) performance status has long been considered a cornerstone of prognosis in patients with advanced cancer.^8^, ^9^ However, this self‐reported assessment is subject to limitations including bias raising doubts on reliability of observations.^10^ Indeed, poor performance status may not be an absolute contraindication to patients with advanced cancer receiving treatments such as immunotherapy, with recent literature reporting that it is safe and tolerable.^11^, ^12^ Furthermore, questioning whether it is a determinant of treatment efficacy.^13^

In contrast to ECOG‐PS, biomarkers of inflammation are generally considered reliable determinants of both treatment effectiveness and likely outcome in patients with advanced cancer.^14^, ^15^ Indeed, biomarkers of systemic inflammation have been reported to have prognostic value in both randomised clinical trials of patients with advanced NSCLC^15^ and in real world clinical practice.^16^ However, with a range of composite scores and cumulative ratios reported within the literature, it remains unclear which ratio/scores should be used. Specifically, if there is heterogeneity in the prevalence and magnitude of systemic inflammation across the different ratios/scores and in their prognostic value to survival.

Composite ratios such as the neutrophil‐lymphocyte ratio (NLR), lymphocyte‐monocyte ratio (LMR) and platelet‐lymphocyte ratio (PLR) have been reported to have prognostic value to clinical outcomes in patients with advanced NSCLC receiving targeted therapies such as tyrosine kinase inhibitors^17^, ^18^ and immunotherapy.^19^, ^20^ However, the ability of such composite measures to discriminate between those who are systemic inflamed from those who are not has previously been questioned.^21^, ^22^, ^23^ Comparison with a sensitive, routinely clinically available measure of the systemic inflammatory response, such as C‐reactive protein (CRP), may be useful method to determine the reliability of such measures using a rational common base.^24^

The aim of the present study was to examine the relationships between systemic inflammatory composite ratios/cumulative scores, the magnitude of the systemic inflammatory response and survival in good performance status patients with advanced, inoperable NSCLC.

## 2 PATIENTS AND METHODS

### 2.1 Patients

Consecutive patients with locally advanced or metastatic NSCLC (TNM stage III or IV disease), who received radiotherapy with palliative intent at the Beatson Oncology Centre, Glasgow, between May 2010 and 2016, were identified from a prospectively maintained database. Patients who were deemed to have good performance status (ECOG‐PS 0/1) and had pre‐treatment bloods facilitating assessment of the systemic inflammatory response were assessed for inclusion. Patients with histologically confirmed small cell lung cancer or those who underwent radiotherapy with curative intent were excluded.

The primary endpoint was overall survival from date of commencing palliative radiotherapy. Date of death was confirmed using hospital electronic records, until the 1st May 2023, which served as the censor date. Ethical approval for this study was granted Greater Manchester East Research Ethics Committee (Rec number: 17/NW/0190). The present study was a retrospective, observational cohort study with no change in patient management. As a result, informed consent was not required in accordance with ethical approval.

### 2.2 Clinicopathological characteristics

Routine demographic details included age, sex and BMI. Age categories were grouped into <64, 65–74 and >74 years. Histological subtype was broadly categorised as adenocarcinoma, squamous cell carcinoma and other/unknown. All tumours were retrospectively staged using the eighth edition of the tumour, node and metastases (TNM) classification and categorised into clinical AJCC stage groupings.^25^ The administration of chemotherapy prior to the patient undergoing radiotherapy and the radiotherapeutic regime administered was identified from hospital electronic records.

### 2.3 Systemic inflammation

Haematological and biochemical results of venous blood samples, obtained within the 6 weeks prior to the patient undergoing radiotherapy, were prospectively recorded. Serum CRP concentration, albumin concentration and differential blood cell counts were used to calculate cumulative scores and composite ratios. The NLR, PLR, LMR and C‐reactive protein albumin ratio (CAR) were all calculated by directly dividing the former by the latter and categorised using thresholds values from the contemporary literature.^21^, ^22^ Similarly, the Neutrophil–lymphocyte score (NLS), Neutrophil–platelet score (NPS), platelet–lymphocyte score (PLS), lymphocyte–monocyte score (LMS) and modified Glasgow Prognostic score (mGPS) were constructed as previously described, using validated threshold values.^21^, ^22^ An auto analyser was used to measure serum CRP (mg/L) and albumin (g/L) concentrations (Architect; Abbot Diagnostics, Maidenhead, UK).

### 2.4 Statistical analysis

The corresponding CRP concentration (mg/L) of each ratio/score category was presented as median (interquartile range, IQR). Demographic data, clinicopathological variables, TNM stage, ECOG‐PS, NLR, LMR, PLR, CAR, NLS, LMS, NPS, PLS, mGPS and overall survival (OS) were presented as categorical variables. Relationships between categorical variables were analysed using the chi‐square test for linear‐by‐linear association.

Univariate and multivariate survival data were analysed using Cox's proportional‐hazards model. Variables associated with OS at a significance level of *p* < 0.1 on univariate analysis were included in multivariate model using a backward conditional approach. OS was defined as the time (months) from the date of commencing palliative radiotherapy to the date of death due to any cause.

Missing data were excluded from analysis on a variable‐by‐variable basis. Two‐tailed *p* < 0.05 were considered statistically significant. Statistical analysis was performed using SPSS software version 28.0. (SPSS Inc., Chicago, IL, USA).

## 3 RESULTS

**FIGURE 1:**
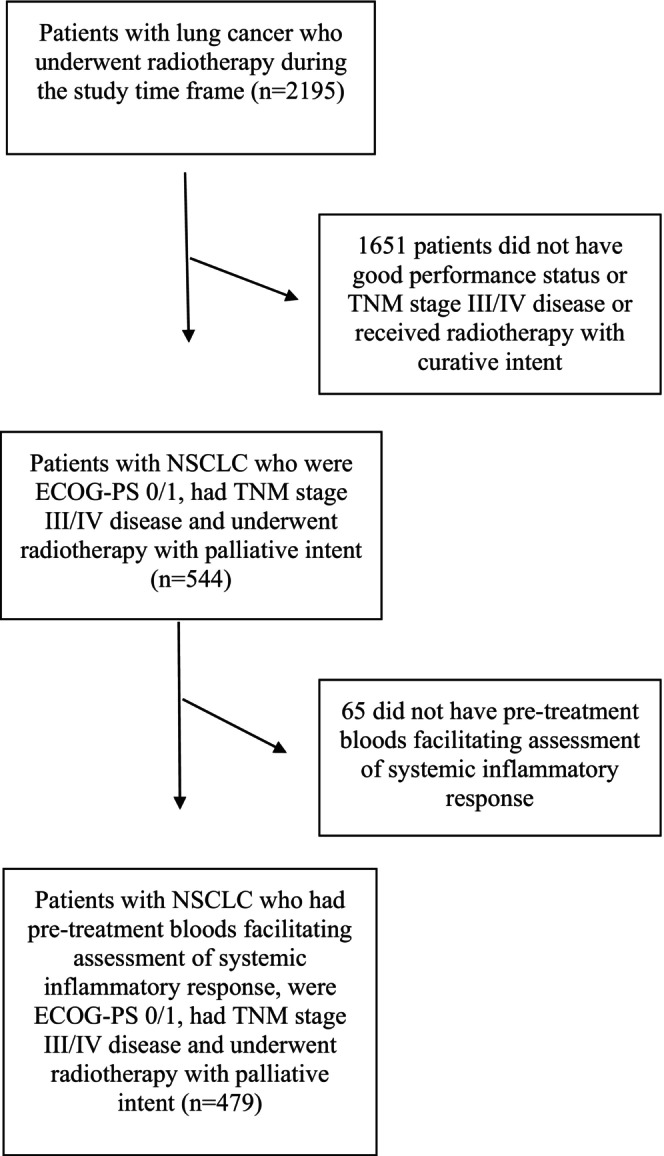
Flow diagram of included patients.

Four hundred and seventy‐nine patients met the inclusion criteria (see Figure 1). The clinicopathological characteristics of the patients included in the study are shown in Table 1. 48% (*n* = 231) of patients were male and 70% (*n* = 338) were ≥65 years of age. 40% (*n* = 190) of patients had an adenocarcinoma and 41% (*n* = 197) had a squamous cell carcinoma. 56% (*n* = 270) of patients had metastatic disease on staging CT‐imaging. 43% (*n* = 208) of patients received chemotherapy prior to undergoing radiotherapy. 39% (*n* = 186) of patients received 36 Gy in 12 fractions, 3% (*n* = 14) of patients received 30 Gy in 10 fractions, 40% (*n* = 191) of patients received 20 Gy in 5 fractions, 9% (*n* = 45) of patients received 8 Gy in 1 fractions and (9% (*n* = 43) of patients received an unknown radiotherapy regime. 29% (*n* = 140) patients were ECOG‐PS 0 and 71% (*n* = 339) were ECOG‐PS 1. 98% (*n* = 469) of patients died during follow‐up. The median survival was 5 months (2–11).

**TABLE 1:** The clinicopathological characteristics of good performance status patients with advanced, inoperable NSCLC receiving palliative radiotherapy.

Variables	*n* = 479 (%)
Age (years)	
<65	141 (30%)
65–74	188 (39%)
>74	150 (31%)
Sex	
Male	231 (48%)
Female	248 (52%)
Histological subtype	
Adenocarcinoma	190 (40%)
Squamous cell	197 (41%)
Unspecified/other	92 (19%)
TNM Stage	
III	209 (44%)
IV	270 (56%)
Chemotherapy	
No	271 (57%)
Yes	208 (43%)
Radiotherapy regime	
36 Gy in 12 fractions	186 (39%)
30 Gy in 10 fractions	14 (3%)
20 Gy in 5 fractions	191 (40%)
8 Gy in 1 fraction	45 (9%)
Unknown	43 (9%)
ECOG‐PS	
0	140 (29%)
1	339 (71%)
Alive	
Yes	10 (2%)
No	469 (98%)

The prevalence and prognostic value of systemic inflammation, across composite ratios and cumulative scores, in good performance status patients with advanced, inoperable NSCLC who received palliative radiotherapy is shown in Table 2. A similar prevalence of systemic inflammation was noted across the various ratios/scores (NLR >3 68%; LMR <2.4 65%; PLR >150 70%; CAR >0.20 83%; NLS ≥1 66%; LMS ≥1 71%; NPS ≥1 50%; PLS ≥1 60% and mGPS ≥1 75%). Furthermore, despite not considered to be systemic inflamed, an NLR <3, LMR ≥2.4, PLR ≤150, NLS 0, LMS 0, NPS 0 and PLS 0 all had a median CRP concentration of >10 m mg/L, generally considered to be inflamed (see Table 2). Finally, on univariate survival analysis, OS was significantly associated with NLR (*p* < 0.001), LMR (*p* < 0.001), PLR (*p* < 0.001), CAR (*p* < 0.001), NLS (*p* < 0.001), LMS (*p* < 0.001), NPS (*p* < 0.001), PLS (*p* < 0.001) and mGPS (*p* < 0.001, see Table 2).

**TABLE 2:** The prevalence and prognostic value of systemic inflammation across composite ratios and cumulative scores in good performance status patients with advanced, inoperable NSCLC who received palliative radiotherapy (*n* = 479).

Inflammation ratio/ score	Ratio/score	Prevalence *n*=/(%)	Hazard ratio (95% CI)	*p*‐Value^a^	Median CRP (mg/L)
Neutropil lymphocyte ratio (NLR)					
Neutrophil count/lymphocyte count	<3	155 (32%)	1.28 (1.15–1.42)	**<0.001**	18 (7–52)
Neutrophil count/lymphocyte count	3–5	199 (42%)			27 (10–57)
Neutrophil count/lymphocyte count	>5	125 (26%)			47 (15–99)
Lymphocyte monocyte ratio (LMR)					
Lymphocyte count/monocyte count	≥2.4	169 (35%)	0.56 (0.46–0.68)	**<0.001**	17 (6–53)
Lymphocyte count/monocyte count	<2.4	310 (65%)			40 (15–87)
Platelet lymphocyte ratio (PLR)					
Platelet count: lymphocyte count	≤150	142 (30%)	1.30 (1.06–1.58)	**0.010**	13 (6–39)
Platelet count: lymphocyte count	>150	337 (70%)			41 (15–84)
C‐reactive protein albumin ratio (CAR)					
C‐reactive protein concentration: albumin concentration	<0.2	65 (17%)	1.31 (1.15–1.50)	**<0.001**	4 (2–6)
C‐reactive protein concentration: albumin concentration	0.2–0.4	61 (15%)			10 (9–13)
C‐reactive protein concentration: albumin concentration	>0.4	269 (68%)			53 (29–97)
Missing		84			
Neutrophil Lymphocyte Score (NLS)					
Neutrophil count ≤7.5 × 10^9^/L and Lymphocyte count ≥1.5 × 10^9^/L	0	165 (34%)	1.36 (1.19–1.55)	**<0.001**	18 (6–46)
Neutrophil count >7.5 × 10^9^/L and Lymphocyte count ≥1.5 × 10^9^/L or Neutrophil count ≤7.5 × 10^9^/L and Lymphocyte count <1.5 × 10^9^/L	1	228 (48%)			38 (13–85)
Neutrophil count >7.5 and Lymphocyte count <1.5 × 10^9^/L	2	86 (18%)			44 (18–109)
Lymphocyte monocyte score (LMS)					
Lymphocyte count ≥1.5 × 10^9^/L and monocyte count ≤0.80 × 10^9^/L	0	139 (29%)	1.39 (1.21–1.60)	**<0.001**	17 (6–45)
Lymphocyte count <1.5 × 10^9^/L and monocyte count ≤0.80 × 10^9^/L or Lymphocyte count >1.5 × 10^9^/L and monocyte count >0.80 × 10^9^/L	1	266 (56%)			36 (13–78)
Lymphocyte count <1.5 × 10^9^/L and monocyte count >0.80 × 10^9^/L	2	74 (15%)			46 (16–121)
Neutrophil Platelet Score (NPS)					
Neutrophil count ≤7.5 × 10^9^/L and platelet count ≤400 × 10^9^/L	0	242 (50%)	1.21 (1.07–1.36)	**0.003**	19 (9–52)
Neutrophil count >7.5 × 10^9^/L and Platelet count ≤400 × 10^9^/L or Neutrophil count ≤7.5 × 10^9^/L and Platelet count >400 × 10^9^/L	1	156 (33%)			35 (12–76)
Neutrophil count >7.5 × 10^9^/L and Platelet count >400 × 10^9^/L	2	81 (17%)			64 (23–111)
Platelet Lymphocyte Score (PLS)					
Platelet count ≤400 × 10^9^/L and Lymphocyte count ≥1.5 × 10^9^/L	0	191 (40%)	1.17 (1.02–1.35)	**0.028**	18 (6–47)
Platelet count >400 × 10^9^/L and Lymphocyte count ≥1.5 × 10^9^/L or Platelet count ≤400 × 10^9^/L and Lymphocyte count <1.5 × 10^9^/L	1	242 (50%)			39 (14–81)
Platelet count >400 × 10^9^/L and Lymphocyte count <1.5 × 10^9^/L	2	46 (10%)			54 (20–122)
Modified Glasgow prognostics score (mGPS)					
CRP ≤10 mg/L and albumin ≥35 mg/L	0	99 (25%)	1.27 (1.12–1.43)	**<0.001**	6 (3–9)
CRP > 10 mg/L and albumin ≥35 mg/L	1	90 (23%)			26 (15–52)
CRP > 10 mg/L and albumin <35 mg/L	2	206 (52%)			58 (29–114)
Missing		84			

*Note*: Bold values are statistically significant.

^a^ *p*‐Value was determined using cox proportional hazards regression.

The correlation between CAR, mGPS and clinicopathological characteristics in good performance status patients with advanced, inoperable NSCLC receiving palliative radiotherapy is shown in Table 3. On univariate analysis, CAR was significantly associated with histological subtype only (*p* < 0.05). On univariate analysis, mGPS was significantly associated with chemotherapy (*p* < 0.05) and ECOG‐PS (*p* < 0.05, see Table 3).

**TABLE 3:** The correlation between CAR and mGPS with clinicopathological characteristics in good performance status patients with advanced, inoperable NSCLC receiving palliative radiotherapy (*n* = 395).

	Age (*n* = 395)	Sex (*n* = 395)	Histological Subtype (*n* = 395)	TNM stage (*n* = 395)	Chemotherapy (*n* = 395)	Radiotherapy regime (*n* = 359)	ECOG‐PS (*n* = 395)
CAR (*n* = 395)	0.671	0.225	**0.048**	0.607	0.152	0.225	0.353
mGPS (*n* = 395)	0.829	0.289	0.176	0.729	**0.019**	0.517	**0.013**

*Note*: Each cell shows *p* value from chi‐square analysis.

Bold values are statistically significant.

The relationship between ECOG‐PS, CAR, mGPS and OS in good performance status patients with advanced, inoperable NSCLC receiving palliative radiotherapy is shown in Table 4. On univariate analysis, CAR>0.40 (*p* < 0.001) and mGPS 2 (*p* < 0.001) were significantly associated with OS. When adjusted for ECOG‐PS, CAR > 0.40 (*p* < 0.001) and mGPS 2 (*p* < 0.05) remained significantly associated with OS (see Table 4).

**TABLE 4:** The relationship between ECOG‐PS, CAR, mGPS and OS in good performance status patients with advanced, inoperable non‐small cell lung cancer receiving palliative radiotherapy (*n* = 395).

	Univariate		Adjusted for ECOG‐PS	
	OS HR (95% CI)	*p*‐Value^a^	OS HR (95% CI)	*p*‐Value^a^
ECOG‐PS (*n* = 395)				
0	‐	‐	‐	‐
1	1.33 (1.06–1.66)	**0.012**	‐	‐
CAR (*n* = 395)				
<0.2	‐	‐	‐	‐
0.2–0.4	1.10 (0.77–1.57)	0.604	‐	‐
>0.4	1.64 (1.25–2.17)	**<0.001**	1.63 (1.24–2.16)	**<0.001**
mGPS (*n* = 395)				
0	‐	‐	‐	‐
1	1.33 (0.99–1.78)	0.055	‐	0.052
2	1.62 (1.27–2.07)	**<0.001**	1.28 (1.02–1.60)	**0.034**

*Note*: Bold values are statistically significant.

^a^ *p*‐Value was determined using cox proportional hazards regression.

## 4 DISCUSSION

The results of the present study show that myeloid‐based systemic inflammation measures such NLR, LMR, PLR, NLS, LMS, NPS, and PLS all capture increasing systemic inflammation as measured using CRP, the prototypical acute phase protein and marker of systemic inflammation and have prognostic value. However, the low‐risk group in each of these measures was associated with CRP concentrations (medians 13–19 mg/L) above the normal range (≤10 mg/L) and therefore these myeloid based measures are not a sensitive measure of systemic inflammation. In contrast, in the CRP based prognostic measures, CAR and mGPS, the low‐risk groups were associated with CRP concentrations (medians 6 and 4 mg/L respectively, within the normal range). The present results, if confirmed in other studies, have important implications for the continued use of myeloid‐based measures of systemic inflammation as prognostic scores in patients with advanced cancer. In particular, how they may be combined with acute phase protein measures of the systemic inflammatory response to enhance prognostic value.^26^

Systemic inflammatory ratios/scores comprised of circulating white blood cells or acute phase proteins represent the systemic responses the bone marrow (myeloid) and liver, respectively.^22^ Both NLR (myeloid) and mGPS (liver) have been reported to have prognostic value to survival in patients with advanced, inoperable NSCLC.^27^, ^28^ However, few studies to date have directly compared the prognostic value of systemic inflammatory composite ratios and cumulative scores in patients with advanced, inoperable NSCLC. As such, the present results are informative. Specifically, that the prevalence of elevated ratio and score varied, with the highest prevalence observed in the acute phase protein‐based CAR and mGPS. This would suggest the greater sensitivity of liver‐based measures of systemic inflammation.

It was of interest that the low‐risk group of all myeloid based inflammatory ratios/scores (NLR <3, LMR ≥2.4, PLR ≤150, NLS 0, LMS 0, NPS 0 and PLS 0) had a median CRP concentration of >10 m mg/L, generally considered to be inflamed (CRP >10 mg/L). While the specificity of myeloid based composite ratios has previously been questioned in studies of primary operable colonic,^22^ rectal^21^ and hepatocellular cancer,^23^ the present study is the first to report this in patients with advanced inoperable cancer. Furthermore, the present results also question the reliability of myeloid based cumulative scores to differentiate those who are inflamed from those who are not. If the present observation were confirmed in future studies, then it would advocate the use of liver‐based measures as the basis to determine the systemic inflammatory status of patients with cancer.^26^

Recent clinical trials have reported that novel target therapies and immunotherapy may improve survival outcomes in patients with NSCLC.^5^, ^6^ Currently, only patients with good performance status patients are generally considered eligible candidates for such treatment.^29^ However, the subjective nature of performance status has implications for the external validity of clinical trials in real world clinical practice.^30^ Furthermore, contemporary studies have challenged the utility of ECOG‐PS to predict likely outcome in patients with advanced NSCLC receiving immunotherapy.^31^ In contrast, liver‐based biomarkers of systemic inflammation, such as mGPS, have been reported to reliably stratify survival in clinical trials of patients with NSCLC.^15^, ^27^ Therefore, it is clear that the mGPS is an objective adjunct to the subjective ECOG‐PS and may be useful in the future allocation of immunotherapy in patients with advanced, inoperable NSCLC.

There are several limitations to the present study. Firstly, this is a single‐centre, retrospective cohort study and has limitations seen with this study design including the potential for sample bias. Secondly, in the present study, when examined in Table 2, the specificity of myeloid measures for quantification of the magnitude of SIR was questioned, with the baseline composite ratios (NLR, PLR, LMR) and baseline cumulative scores (NLS, PLS, LMS, NPS) all having a median CRP concentration >10 mg/L (generally considered systemically inflamed). Therefore, only the prognostic value of liver‐based measures of systemic inflammation (CAR and mGPS) was examined in relation to overall survival, adjusted for ECOG‐PS.^8^ Nevertheless, given their extensively reported prognostic value and provided that their baseline threshold was associated with a CRP ≤10 mg/L, comparison of the prognostic value of these composite ratios (NLR, PLR, LMR) and cumulative scores (NLS, PLS, LMS, NPS) would be of considerable interest. Thirdly, the patients included in the present study were recruited between 2010 and 2016. As such, few patients were likely to have received immunotherapy or targeted therapies which are now recognised to have a profound impact on survival in a proportion of patients.^29^ However, it of interest that systemic inflammation based prognostic scores are now recognised to prognostic value in such targeted therapies and immunotherapy.^32^, ^33^ Therefore, the present results are likely to be relevant for current clinical practice. Lastly, in the present cohort included only good performance status patients (ECOG‐PS 0/1). Further study of larger cohorts including poor performance status patients (in particular ECOG‐PS 2) are required to determine whether systemic inflammatory biomarkers stratify survival of patients with advanced, inoperable NSCLC in real world clinical practice.

In summary, compared with myeloid based measures of the systemic inflammatory response, liver‐based measures (CAR and mGPS) appear to be more reliable in quantifying the magnitude of the systemic inflammatory response and therefore should form the basis of systemic inflammation based prognostic scores in patients with advanced NSCLC.

## AUTHOR CONTRIBUTIONS

**Josh McGovern:** Conceptualization (lead); data curation (lead); formal analysis (lead); writing – original draft (lead); writing – review and editing (lead). **Fraser O'Rourke:** Data curation (equal). **Sarah Will:** Data curation (equal). **Hanh Thi Ngoc Nguyen:** Writing – review and editing (equal). **Elise Cranfield:** Data curation (equal). **Charlotte Maseland:** Data curation (equal). **Nicholas MacLeod:** Data curation (equal); writing – review and editing (equal). **John D. Maclay:** Data curation (equal); writing – review and editing (equal). **Barry J. Laird:** Writing – review and editing (equal). **Ross D. Dolan:** Data curation (equal). **Donald C. McMillan:** Conceptualization (lead); formal analysis (lead); methodology (supporting); writing – original draft (lead); writing – review and editing (lead). The authors would like to Randa Saeed for her assistance with the study.

## CONFLICT OF INTEREST STATEMENT

No funding or conflicts of interest to declare.

## Data Availability

The datasets generated and/or analysed during the current study are not publicly available but are available from the corresponding author on reasonable request.
